# Neutrophil-to-High-Density Lipoprotein Ratio (NHR) and Neutrophil-to-Lymphocyte Ratio (NLR) as prognostic biomarkers for incident cardiovascular disease and all-cause mortality: A comparison study

**DOI:** 10.1016/j.ajpc.2024.100869

**Published:** 2024-10-12

**Authors:** Shih-Ming Chuang, Sung-Chen Liu, Ming-Nan Chien, Chun-Chuan Lee, Yuan-Teh Lee, Kuo-Liong Chien

**Affiliations:** a Institute of Epidemiology and Preventive Medicine, College of Public Health, National Taiwan University, Taipei, Taiwan; b Division of Endocrinology and Metabolism, Department of Internal Medicine, Mackay Memorial Hospital, Taipei, Taiwan; c Department of Medicine, Mackay Medical College, Taipei, Taiwan; d Mackay Junior College of Medicine, Nursing, and Management, Taipei, Taiwan; e Department of Internal Medicine, National Taiwan University Hospital, Taipei, Taiwan

**Keywords:** Cardiovascular diseases, Mortality, Neutrophil-to-lymphocyte ratio (NLR), Neutrophil-to-HDL cholesterol ratio (NHR)

## Abstract

Cardiovascular diseases (CVD) remain a leading cause of global mortality, with atherosclerosis and inflammation playing pivotal roles in their development. The neutrophil-to-lymphocyte ratio (NLR) and neutrophil-to-HDL cholesterol ratio (NHR) have emerged as potential biomarkers for assessing CVD risk. In this community-based cohort study conducted in Taiwan, involving 3278 participants, we investigated the associations between NHR, NLR, and the risks of CVD and all-cause mortality. Our findings revealed that both NHR and NLR were effective in identifying individuals at high risk for CVD. However, when assessing their joint effect, NHR alone demonstrated a stronger predictive value for CVD prognosis than NLR or the combination of both markers. Furthermore, NLR alone showed potential as a predictor of all-cause mortality when compared with NHR alone or in combination with NLR and NHR. These findings underscore the complex interplay between inflammation and lipid metabolism in the pathogenesis of CVD. While NHR shows promise as a cost-effective tool for CVD risk assessment, NLR emerges potential as a prognostic marker for mortality. Further research is warranted to explore the dynamic changes in these markers and their implications for clinical practice.

## Highlights

• The study examines NHR and NLR as biomarkers for predicting cardiovascular disease (CVD) and all-cause mortality.
• Both NHR and NLR are effective in identifying high CVD risk, with NHR showing stronger predictive value than NLR or their combination.
• NLR alone is a stronger predictor of all-cause mortality than NHR or the combination of NHR and NLR.
• The study explores the roles of neutrophils, lymphocytes, and HDL in inflammation, lipid metabolism, and atherosclerosis.
• Clinical implications: NHR is a cost-effective tool for CVD risk, while NLR is more relevant for mortality; their combination adds no benefit for CVD or mortality outcomes.

## Graphical abstract

**Figure:**
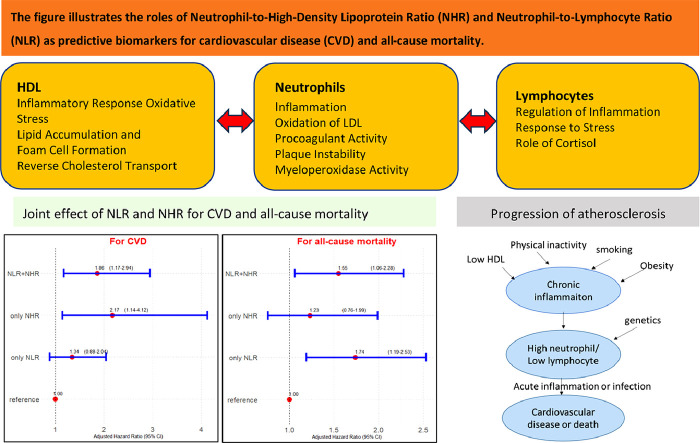

## 1 Introduction

Cardiovascular diseases (CVD), which encompass conditions such as coronary artery disease (CAD) and stroke, are the primary contributors to worldwide mortality, morbidity, and disability-adjusted life-years, accounting for approximately one-third of all global deaths [1]. This significant impact highlights their crucial role in the field of public health. While numerous factors can contribute to the development of CVD, a predominant cause is atherosclerosis, a condition that arises from the accumulation of plaque on the walls of arteries. Past research has established a connection between the progression of atherosclerosis and inflammation [2].

The development of CVD is influenced by inflammation and disrupted lipid metabolism. Immune cells focus on the white blood count, including neutrophils, lymphocytes, and monocytes, and can trigger the rupture of atherosclerotic plaques, ultimately resulting in CVD [3]. A strong correlation between the occurrence of cardiac adverse events and a high abundance of neutrophils has been shown [4]. In the last decade, research on the neutrophil-to-lymphocyte ratio (NLR) as a disease marker has gained traction. Although a specific cut-off value has not been identified, the role of NLR as an indicator of immune system balance is well established. It serves as a prognostic marker, independently correlating with mortality in various conditions, including sepsis, pneumonia, COVID-19, cancer, and CVD [5].

Elevated low-density lipoprotein (LDL) cholesterol levels also contribute to the risk of atherosclerosis, while high-density lipoprotein (HDL) cholesterol plays a protective role in this context [6]. An inverse relationship between HDL-C levels and the risk of CAD has been indicated in recent studies, suggesting its potential as a strong predictor of CAD prognosis [6]. An emerging parameter, the neutrophil-to-HDL-cholesterol ratio (NHR), is gaining attention in cardiovascular disease research as it is designed to provide a more comprehensive assessment of inflammatory status and lipid metabolism. The NHR is believed to be a cost-effective and emerging biomarker for systemic inflammation, demonstrating its screening in CVD [7]. The NHR is also used for risk stratification and the prediction of both short- and long-term outcomes in CVD [8]. The potential application of this measure represents an inexpensive, easily accessible, and minimally invasive blood-based measure, making it particularly valuable in regions with limited resources.

In clinical practice, NHR and NLR are not only considered as one component of the white blood cell count but also as cost-effective biomarkers for systemic inflammation. In recent years, they have been associated with the occurrence and prognosis of many diseases [[9], [10], [11]]. Previous epidemiological studies demonstrated that elevated levels of individual NHR is associated not only with increased CVD risk but also with higher all-cause mortality rates [12]. While the recent application of NHR and NLR in the context of CVD and mortality has gained significant attention, to the best of our knowledge, there has been no study comparing the significance of the effects of NHR and NLR on the outcomes of CVD and all-cause mortality. Therefore, the primary aim of our research was to investigate the associations between NHR and NLR and their impact on CVD and all-cause mortality within a community-based cohort study conducted in Taiwan.

## 2 Methods

### 2.1 Study design and participants

From 1990 to 2005, all participants residing in the Chin-Shan community were recruited for the Chin-Shan Community Cardiovascular Cohort study (CCCC). This study followed a prospective cohort design, and detailed information about the current cohort study can be found in previous publications [13,14]. A total of 3602 participants, consisting of 47.3 % men and 52.7 % women, all over the age of 35 at the beginning of the study, were included. This study received approval from the Research Ethics Committee of the National Taiwan University Hospital (Approval Number: 202108099RINA). Informed consent was obtained from all participants or their relatives, who signed the consent forms before enrollment. All methods were conducted in accordance with the relevant guidelines and regulations.

Since the enrollment of subjects in the trial, all participants underwent individual assessments through the administration of questionnaires and personal interviews. Participants invited to join this study were scheduled for physical examinations and laboratory investigations conducted by physicians.

Blood samples were collected from participants to measure serum biochemical indices, including blood glucose, lipid profiles, liver function, and kidney function(estimated Glomerular filtration rate). The laboratory measurements in this study were conducted at different time points: initial plasma biochemical biomarkers were measured at baseline in 1993, and C-reactive protein (CRP) levels were additionally measured in 1994. These measurements were repeated during the subsequent follow-up period until 2015.

### 2.2 NLR and NHR for exposure

NHR or NLR is determined by dividing the neutrophil count by the HDL-C value or by dividing the neutrophil count by the lymphocyte count, respectively. The absolute neutrophil and lymphocyte counts were acquired from The UniCel DxH 800 Analyzer. We utilized the Roche modular P and Roche Cobas 6000 chemistry analyzers to assess HDL-C levels. We completed the collection of blood samples according to the established blood collection procedures. In addition, as the blood samples were not sent to the clinical laboratory of National Taiwan University Hospital within 8 h after collection, we planned to place them in liquid nitrogen at −76 °C. The association between NHR or NLR and the desired outcomes was assessed based on the distribution of quartiles of exposure.

### 2.3 Outcome variables

The outcomes of interest in this investigation encompassed both CVD and all-cause mortality. The definitions of CVD events considered in this study included patients with incident CAD or stroke. Incident CAD was characterized by nonfatal myocardial infarction, fatal coronary heart disease, and hospitalization due to percutaneous coronary intervention and coronary bypass surgery. The incidence of stroke included ischemic, hemorrhagic, and unclassified types. All-cause mortality was identified through hospital records or the government vital registry.

### 2.4 Statistical analysis

NHR values were stratified into quartile ranges: quartile 1 (<2.43), quartile 2 (2.43–3.32), quartile 3 (3.32–4.49), and quartile 4 (>4.49). Similarly, NLR values were categorized into quartile ranges: quartile 1 (<1.35), quartile 2 (1.35–1.73), quartile 3 (1.73–2.30), and quartile 4 (>2.30). Continuous variables were expressed as means and standard deviations (SD) or medians assessed by the Wilcoxon rank-sum test, or ANOVA was applied to confirm statistical significance. Categorical variables presented as frequency and percentage were calculated by the chi-square method. We adopted the Receiver Operating Characteristic (ROC) curve to calculate the Area Under the Curve (AUC) for evaluating the NLR and NHR classification model in CVD and all-cause mortality. Additionally, we determined the cut-off that offers the optimal balance between sensitivity and specificity based on the Youden Index.

The Cox proportional hazard model was used to estimate the adjusted hazard ratios (HR) of NHR and NLR on the risk of unfavorable outcomes. We used three models to explore the relations: model 1 was adjusted for sex and age; model 2 was adjusted for model 1 covariates plus body mass index, smoking, hypertension, dyslipidemia, and diabetes; and model 3 was adjusted for model 2 covariates plus systolic blood pressure, triglycerides, LDL, and white blood cells. The spline-based HR model was used to evaluate the effect of the continuous variable of interest of NHR and NLR levels on the risk of incident adverse outcomes. The cutoff values for NHR and NLR were determined based on the results of the ROC curve. All patients were divided into four groups. CVD and mortality were stratified for analysis using Cox regression. Additionally, we analyzed the interaction between NHR and NLR. To assess collinearity in the prediction models involving NLR and NHR, we conducted variance inflation factor (VIF) analysis, considering potential issues due to their shared neutrophil component. To mitigate bias and enhance model accuracy, we also explored alternative ratios such as 1/HDL and 1/lymphocyte, which do not share the same component, aiming to improve the stability and interpretability of the results.

## 3 Results

**Fig. 1:**
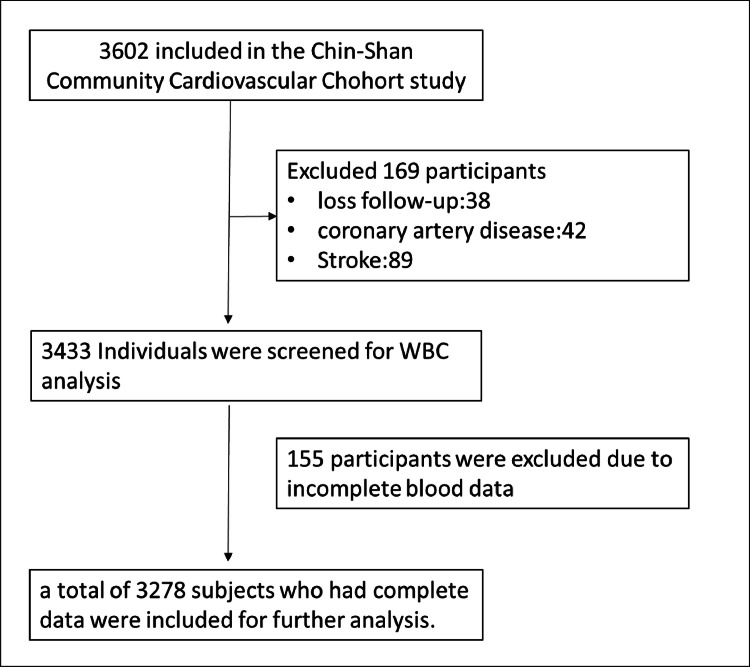
The flow diagram of participants.

To assess the screening capabilities of NHR and NLR for long-term CVD events in this study, we excluded individuals who were lost to follow-up (*n* = 38), as well as those who had pre-existing CAD (*n* = 42), baseline stroke (*n* = 89), or incomplete blood data (*n* = 155) from the study during follow-up. A total of 3278 participants who completed all examinations were included for further analysis (see Fig. 1). Over a median follow-up period of 12.6 years, a total of 323 participants developed ASCVD, and 728 participants died.

The baseline characteristics of all participants were classified according to the quartiles of NHR and NLR, as shown in Table 1, Table 2. The average age was 54.9 ± 12.4 years. Compared to the NHR quartile group, patients in the high NHR quartile were significantly older, had a higher proportion of smokers, and were predominantly male (all *P* < 0.05); furthermore, elevated blood pressure and BMI were observed. The laboratory figures for glucose, triglycerides, LDL, uric acid, blood urea nitrogen, creatinine, white blood cell count, neutrophil count, NHR, and NLR were higher in the high NHR quartile group, while HDL was significantly lower (all *P* < 0.05). Except for triglycerides, LDL, HDL, blood glucose, and blood pressure, the baseline characteristics were similar between the NHR and NLR groups.

**Table 1:** Characters of subjects according to quartile of Neutrophil-to-HDL ratio (NHR)

	Q1(<2.43)			Q2(2.43–3.32)			Q3(3.32–4.49)			Q4(>4.49)			Total			p
N	802			810			808			811			3231			
SEX (women,%)	64.2			58.3			52.7			40.8			54.0			<0.001
Age(year)	54.1	±	11.9	54.8	±	12.1	54.9	±	12.6	55.9	±	12.5	54.9	±	12.3	0.047
BMI(kg/m^2^)	22.5	±	3.2	23.2	±	3.2	23.8	±	3.4	24.4	±	3.5	23.5	±	3.4	<0.001
Smoking (%)	22.4	27.3	30.0	42.0	30.5	<0.001										
BUN(mg/dL)	18.1	±	6.6	18.4	±	6.4	18.6	±	6.8	19.3	±	7.1	18.6	±	6.7	0.003
Cr(mg/dL)	0.8	±	0.5	0.8	±	0.3	0.8	±	0.3	0.9	±	0.4	0.9	±	0.4	<0.001
Uric acid	5.2	±	1.5	5.5	±	1.7	5.7	±	1.7	6.1	±	1.7	5.6	±	1.7	<0.001
TC(mg/dL)	197.7	±	45.5	195.8	±	44.5	198.3	±	43.4	198.5	±	46.6	197.6	±	45.0	0.631
TG(mg/dL)	90.2	±	59.9	106.8	±	66.3	128.8	±	85.7	172.2	±	124.4	125.6	±	93.4	<0.001
GOT(mg/dL)	36.3	±	19.4	36.1	±	21.8	35.7	±	25.2	35.7	±	16.9.	35.9	±	21.1	0.956
GPT(mg/dL)	45.0	±	24.5	45.8	±	21.2	46.4	±	29.2	49.8	±	23.2	46.8	±	24.7	<0.001
GLU(mg/dL)	104.9	±	23.2	107.1	±	27.1	111.5	±	31.6	117.7	±	40.3	110.2	±	31.8	0.001
HDL(mg/dL)	56.7	±	11.3	50.0	±	9.9	44.8	±	9.3	38.4	±	8.3	47.2	±	11.7	<0.001
LDL(mg/dL)	129.7	±	44.5	134.0	±	42.4	140.6	±	42.0	147.2	±	44.4	138.1	±	43.8	<0.001
SBP(mmHg)	122.8	±	19.3	124.1	±	20.1	126.5	±	21.2	128.4	±	20.6	125.5	±	20.4	<0.001
DBP(mmHg)	76.0	±	10.2	76.0	±	11.3	77.4	±	11.6	79.4	±	11.0	77.2	±	11.1	<0.001
WBC(10^9^/L)	4.9	±	0.9	5.7	±	0.9	6.5	±	1.2	7.6	±	1.4	6.27	±	1.69	<0.001
Neutrophils (10^9^/L)	2.8	±	0.6	3.6	±	0.7	4.3	±	0.9	5.2	±	1.2	4.0	±	1.3	<0.001
Lymphocytes (10^9^/L)	2.2	±	2.8	2.3	±	2.0	2.3	±	0.7	2.4	±	0.7	2.3	±	1.8	0.094
NHR	1.9	±	0.4	2.9	±	0.3	3.8	±	0.3	6.0	±	0.3	3.7	±	2.0	<0.001
NLR	1.4	±	0.6	1.8	±	0.8	2.1	±	0.8	2.5	±	1.1	1.9	±	1.0	<0.001
CRP	0.2	±	0.4	0.2	±	0.5	0.3	±	0.5	0.3	±	0.5	0.3	±	0.5	0.209
eGFR	90.6	±	19.6	88.9	±	20.6	89.0	±	20.1	86.8	±	21.5	88.8	±	20.5	0.002
Stain (%)	7.0	8.7	11.8	17.5	11.4	<0.001										

Data are presented as mean value ± standard deviation or %.

BMI=body mass index; Cr=creatinine; URCA=uric acid; TC=total cholesterol; TG=triglyceride; GPT=glutamic-pyruvic transaminase; GLU= fasting plasma glucose; PPG=post-prandial plasma glucose; HbA1c=glycosylated hemoglobin; LDL-C= low-density lipoprotein cholesterol; HDL-C= high-density lipoprotein cholesterol; SBP=systolic blood pressure; DBP= diastolic blood pressure; WBC=white blood cell; NHR= Neutrophil-to-HDL ratio; NLR= Neutrophil-to-lymphocyte ratio; CRP= C-Reactive Protein; eGFR= estimated Glomerular filtration rate; *Q*=quartile.

**Table 2:** Characters of subjects according to quartile of Neutrophil to lymphocyte ratio(NLR).

	Q1(<1.35)			Q2(1.35–1.73)			Q3(1.73–2.30)			Q4(>2.30)			Total			P
N	809			799			858			812			3278			
SEX (women,%)	60.6			53.4			54.8			47.0			53.9			<0.001
Age(year)	53.83	±	12.01	53.86	±	11.90	54.80	±	12.68	56.97	±	12.53	54.87	±	12.35	<0.001
BMI(kg/m^2^)	23.43	±	3.39	23.76	±	3.45	23.60	±	3.38	23.17	±	3.52	23.49	±	3.44	0.004
Smoking (%)	25.7	31.1	30.6	34.6	30.5	<0.001										
BUN(mg/dL)	17.89	±	5.91	18.34	±	6.38	18.52	±	6.85	19.79	±	7.60	18.63	±	6.75	<0.001
Cr(mg/dL)	0.84	±	0.26	0.83	±	0.27	0.85	±	0.45	0.89	±	0.40	0.85	±	0.36	0.007
Uric acid	5.55	±	1.68	5.69	±	1.65	5.55	±	1.58	5.82	±	1.86	5.65	±	1.70	0.002
TC(mg/dL)	199.79	±	46.31	196.73	±	44.74	197.01	±	45.20	198.36	±	45.07	197.96	±	45.33	0.505
TG(mg/dL)	124.99	±	91.73	131.49	±	104.08	126.22	±	96.88	121.80	±	87.30	126.11	±	95.21	0.230
GOT(mg/dL)	37.32	±	19.26	36.16	±	25.28	35.34	±	18.42	36.26	±	24.47	36.25	±	22.01	0.335
GPT(mg/dL)	48.28	±	26.44	48.19	±	31.37	46.53	±	25.07	44.99	±	18.31	46.99	±	25.72	0.030
GLU(mg/dL)	109.55	±	27.47	111.01	±	33.49	109.10	±	30.49	110.99	±	34.47	110.14	±	31.58	0.497
HDL(mg/dL)	48.13	±	12.68	46.96	±	11.99	46.43	±	12.20	48.45	±	13.49	47.48	±	12.62	0.003
LDL(mg/dL)	139.40	±	45.51	136.98	±	43.21	138.10	±	43.90	136.82	±	42.91	137.83	±	43.89	0.622
SBP(mmHg)	125.08	±	19.79	124.75	±	18.99	125.39	±	21.27	126.83	±	21.38	125.52	±	20.41	0.178
DBP(mmHg)	77.36	±	10.88	77.30	±	10.90	76.66	±	11.34	77.44	±	11.47	77.18	±	11.15	0.453
WBC(10^9^/L)	5.72	±	1.48	6.07	±	1.48	6.29	±	1.54	7.01	±	1.97	6.27	±	1.69	<0.001
Neutrophils (10^9^/L)	2.9	±	08	3.7	±	0.9	4.2	±	1.0	5.4	±	2.1	4.0	±	1.6	<0.001
Lymphocytes(10^9^/L)	2.9	±	3.2	2.4	±	0.6	2.1	±	0.5	1.8	±	0.5	2.3	±	1.7	<0.001
NHR	2.6	±	1.1	3.4	±	1.3	3.9	±	1.6	4.8	±	2.9	3.7	±	2.0	<0.001
NLR	1.1	±	0.2	1.5	±	0.1	2.0	±	0.2	3.2	±	1.1	1.9	±	1.0	<0.001
CRP	0.3	±	0.6	0.2	±	0.3	0.3	±	0.6	0.3	±	0.6	0.3	±	0.6	0.619
eGFR	90.3	±	20.7	89.1	±	20.7	88.6	±	19.7	86.6	±	21.2	88.6	±	20.6	0.005
Stain (%)	9.8	10.4	10.5	15.3	11.5	0.001										

Data are presented as mean value ± standard deviation or %.

BMI=body mass index; Cr=creatinine; URCA=uric acid; TC=total cholesterol; TG=triglyceride; GPT=glutamic-pyruvic transaminase; GLU= fasting plasma glucose; PPG=post-prandial plasma glucose; HbA1c=glycosylated hemoglobin; LDL-C= low-density lipoprotein cholesterol; HDL-C= high-density lipoprotein cholesterol; SBP=systolic blood pressure; DBP= diastolic blood pressure; WBC=white blood cell; NHR= Neutrophil-to-HDL ratio; NLR= Neutrophil-to-lymphocyte ratio; CRP= C-Reactive Protein; eGFR= estimated Glomerular filtration rate; *Q*=quartile.

The univariate Cox regression analyses revealed that the hazard ratio (HR) for developing CVD was 1.47 (95 % CI 1.01–2.12) for NHR quartile 3 and 2.20 (95 % CI 1.56–3.10) for NHR quartile 4. Notably, individuals in the high NHR group in quartile 4 exhibited a significantly elevated risk of developing CVD, with an HR of 1.74 (95 % CI 1.05–2.88), even after adjustment for age, sex, hypertension, diabetes, LDL, fasting glucose, renal function CRP, estimated Glomerular filtration rate, statin use, antihypertensive agents, systolic blood pressure, and the white blood cell count. Regarding NLR and its association with the risk of CVD, a high NLR in quartile 4 remained significantly associated with CVD even after adjustment for relevant confounding factors.

For all-cause mortality, univariate Cox regression analyses indicated that the highest NHR quartile was associated with an elevated risk of mortality (HR for quartile 4 vs. quartile 1, 1.48; 95 % CI: 1.20–1.83). However, this finding did not reach statistical significance after adjustment for confounding factors (adjusted HR for quartile 4 vs. quartile 1, 1.04; 95 % CI: 0.72–1.45). By contrast, after adjusting for confounders, the high NLR quartile not only remained associated with mortality but also retained statistical significance (adjusted HR for quartile 4 vs. quartile 1, 1.62; 95 % CI: 1.14–2.30) (see Table 3).

**Table 3:** Risk of cardiovascular disease or all-cause d of participants associated with NHR and NLR.

	CVD				All-cause mortality			
NHR								
	Crude		adjusted		Crude		adjusted	
Varialbes	HR (95 % CI)	P-value	HR (95 % CI)	P-value	HR (95 % CI)	P-value	HR (95 % CI)	P-value
Per SD increment of NHR	1.29(1.14–1.45)	<0.001	1.21(1.10–1.32)	<0.001	1.10(1.02–1.18)	0.016	1.15(1.07–1.23)	<0.001
Quartile								
Q1(<3.16)	1	Ref.	1	Ref.	1	Ref.	1	Ref.
Q2(3.16–5.02)	1.28(0.87–1.87)	0.208	1.21(0.70–2.10)	0.493	1.12(0.89–1.40)	0.327	0.96(0.70–1.38)	0.826
Q3(5.02–7.92)	1.47(1.01–2.12)	0.043	1.23(0.72–2.10)	0.441	1.20(0.96–1.50)	0.104	0.97(0.76–0.1.24)	0.842
Q4(>7.92)	2.20(1.56–3.10)	<0.001	1.74(1.05–2.88)	0.030	1.48(1.20–1.83)	<0.001	1.04(0.72–1.45)	0.911
P for trend		<0.001		0.022		<0.001		0.088
NLR								
Per SD increment of NLR	1.18(1.05–1.33)	0.005	1.14(1.02–1.27)	0.022	1.32(1.22–1.42)	<0.001	1.21(1.14–1.29)	<0.001
Quartile								
Q1(<1.35)	1	Ref.	1	Ref.	1	Ref.	1	Ref.
Q2(1.35–1.73)	1.33(0.94–1.89)	0.112	1.27(0.89–1.82)	0.185	1.16(0.92–1.46)	0.219	1.27(0.89–1.87)	0.173
Q3(1.73–2.30)	1.38(0.98–1.95)	0.065	1.30(0.92–1.84)	0.140	1.22(0.97–1.53)	0.089	1.33(0.93–1.89)	0.117
Q44(>2.30)	1.67(1.18–2.35)	0.003	1.44(1.02–2.04)	0.039	2.11(1.71–2.60)	<0.001	1.62(1.14–2.30)	0.007
P for trend		0.005		0.047		<0.001		0..006

NHR= neutrophil-to-HDL ratio; NLR=neutrophil-to-lymphocyte ratio; CAD= cardiovascular disease; *Q*=quartile.

Adjusted for age, sex, hypertension, diabetes, LDL, fasting glucose, C-Reactive Protein, estimated Glomerular filtration rate , statin use, antihypertension agents, systolic blood pressure, white blood cell.

**Fig. 2:**
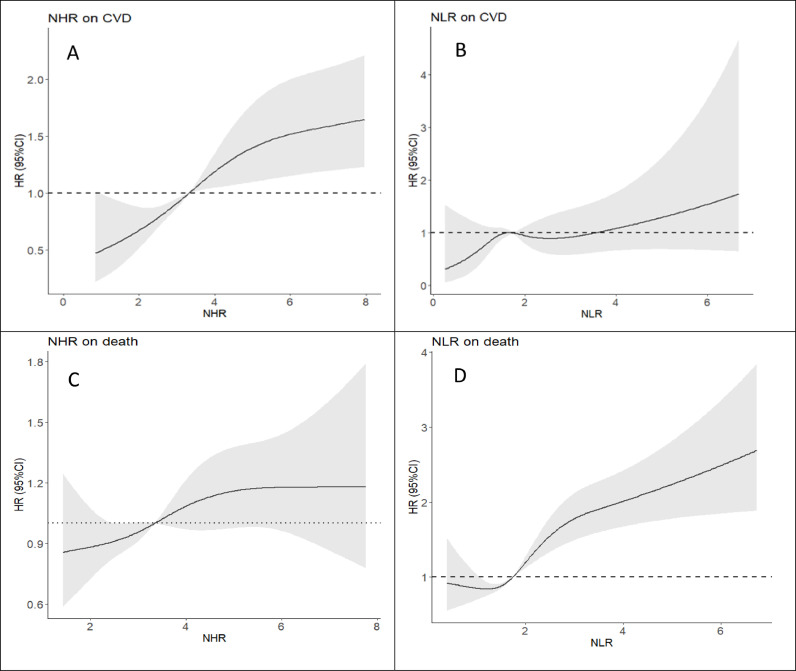
Association of NHR (A) and NLR (B) level to the risk of CVD and NHR(C) NLR(D) level to the risk of all-cause mortality on spline regression model.

**Fig. 3:**
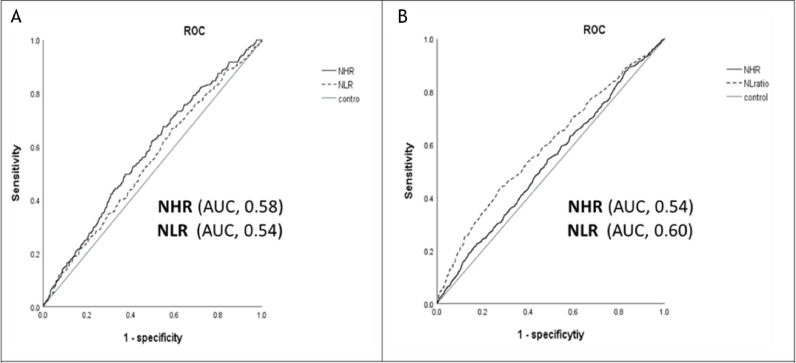
ROC curves of NHR and NLR for CVD (A) and all-cause mortality (B).

Spline regression analysis revealed a dose-response relationship between both NHR and NLR levels and CVD. However, only NLR exhibited a dose-response relationship with the risk of mortality, whereas NHR did not demonstrate such a pattern (Fig. 2). In our analysis of the receiver operating characteristic (ROC) curve, we determined the most effective threshold for distinguishing cases related to CVD or mortality. For CVD, the threshold of NHR was identified as 4.55, providing a sensitivity of 67.5 % and a specificity of 45.2 % (AUC, 0.58; 95 %CI = 0.55– 0.62, *P* < 0.001). Similarly, NLR was established as the optimal threshold for CVD, determined to be 1.60, with a sensitivity of 65.3 % and a specificity of 41.8 % (AUC, 0.54; 95 %CI = 0.50–0.58, *P* < 0.029). For mortality, NHR had a threshold of 3.37 with 54.6 % sensitivity and 52.8 % specificity (AUC 0.54; 95 % CI 0.52–0.57, *P* = 0.011). The optimal mortality threshold of NLR was 2.13, yielding a sensitivity of 43.1 % and a specificity of 72.5 % (AUC 0.60; 95 % CI 0.57–0.63, *P* < 0.001) (see Fig. 3).

We assessed the joint effect of the two markers by dividing the participants into four groups using the appropriate thresholds of NHR and NLR for CVD and mortality outcomes (Table 4). Although a high risk of CVD was observed in the group with a combined high NHR and NLR (NHR > 4.55 and NLR > 1.6), the highest risk was observed for those with a high NHR alone (NHR > 4.55) in the stratified analysis conducted using the Cox proportional hazard model (adjusted HR, 2.17; 95 % CI: 1.14–4.12; *P* = 0.019). For the mortality outcome, a similar pattern was observed, where the group with NLR alone exhibited the highest risk of mortality, followed by the combined NHR and NLR group with the second highest risk (adjusted HR 1.74; 95 % CI: 1.19–2.53 vs. 1.55; 95 % CI: 1.06–2.28). There was no evident interaction detected between the impacts of NHR and NLR on the risk of CVD and mortality outcomes.

**Table 4:** Joint effects of NHR and NLR on` cardiovascular disease or all-cause mortality.

CVD							
NHR>4.55	NLR>1.6	N	Case	crude		Adjusted	
				HR (95 % CI)	P-value	HR (95 % CI)	P-value
No	No	806	49	Ref.		Ref.	
No	Yes	617	42	1.50(1.12–2.01)	0.007	1.34(0.88–2.04)	0.167
Yes	No	518	46	2.61(1.58–4.32)	<0.001	2.17(1.14–4.12)	0.019
Yes	Yes	1288	143	2.12(1.51–2.97)	<0.001	1.86(1.17–2.94)	0.008
Interaction of NHR and NLR			1.00(0.99–1.01)	0.639	0.99(0.95–1.03)	0.652	

All-cause mortality							
NHR>3.37	NLR>2.13	N	Case	crude	Adjusted	Crude	Adjusted
				HR (95 % CI)	P-value	HR (95 % CI)	P-value
No	No	1310	214	Ref.		Ref.	
No	Yes	312	101	2.19(1.73–2.78)	<0.001	1.74(1.19–2.53)	0.004
Yes	No	906	175	1.19(0.97–1.45)	0.092	1.23(0.76–1.99)	0.395
Yes	Yes	701	200	1.83(1.50–2.23)	<0.001	1.55(1.06–2.28)	0.024
Interaction of NHR and NLR			1.00(1.00–1.01)	0.023	1.01(0.98–1.03)	0.580	

NHR= neutrophil-to-HDL ratio; NLR= neutrophil-to-lymphocyte ratio; CVD=cardiovascular diseases.

Adjusted for age, sex, hypertension, diabetes, LDL, fasting glucose, C-Reactive Protein, estimated Glomerular filtration rate , statin use, antihypertension agents, systolic blood pressure, white blood cell.

The VIF analysis indicated that there were no significant collinearity issues between NLR and NHR. However, to further reduce potential bias, we used alternative ratios such as 1/HDL and 1/lymphocyte. This approach improved the predictive accuracy and stability of the models, and even when considering ratios that do not share the same neutrophil component, the robust results remained consistent.

## 4 Discussion

This prospective and observational cohort study investigated the impact of NHR and NLR on the risk of CVD and all-cause mortality. Our findings revealed that both NHR and NLR are effective in identifying individuals with an elevated risk of CVD. However, upon evaluating the joint effect of NHR and NLR, we observed that NHR alone holds superior predictive value for the prognosis of CVD compared to NLR alone or their combination. For the assessment of mortality risk, NLR alone emerges as more relevant than either NHR alone or the combination of NLR and NHR. Therefore, NLR demonstrates the potential to predict all-cause mortality.

CVD mainly arises from arteriosclerosis, with inflammation and lipid metabolism disorder playing crucial roles in its development [15,16]. Earlier studies pointed to a robust association between the levels of neutrophils, lymphocytes, and HDL and the onset of atherosclerosis and major adverse cardiovascular events (MACE) [17,18]. Currently, multiple studies suggest that a combination of inflammation and lipids may provide a more thorough representation of CVD prognosis when compared to a single lipid alone. Involving a complex interaction between lipids and immune-inflammatory cells, atherosclerosis has been demonstrated in studies to activate the coagulation system through substances derived from inflammatory cells. This activation occurs even in the absence of conventional risk factors, underscoring the crucial role of dysregulation in the inflammatory system for pathological thrombosis [19]. As a precursor to the inflammatory process, recent research suggests that the pivotal regulatory role of neutrophils in cardiovascular inflammation has gained significance in CVD due to its association with various stages of atherosclerosis [20,21].

### 4.1 Neutrophils and atherosclerosis

After LDL is oxidized, it is engulfed by monocytes, forming foam cells that deposit within the vascular intima, leading to the development of atherosclerotic plaques. Elevated neutrophil levels caused by hypercholesterolemia are positively associated with early atherosclerosis development [22]. Neutrophils contribute to thrombosis and subsequent CVD through coagulation initiation, platelet activation, and the secretion of procoagulant granzymes [23]. This link can influence the formation of atherosclerotic plaques and trigger thrombotic complications associated with atherosclerosis. However, neutrophils play a dual role in cardiovascular inflammation, as they have both reparative and eliminative functions at the site of myocardial damage [24]. Plaque destabilization arises from neutrophil infiltration into atherosclerotic plaques, accompanied by the release of various proteolytic enzymes, including neutrophil elastase, by activated neutrophils. This process facilitates basement membrane deterioration and endothelial dysfunction [25]. Neutrophils have been associated with the development of CVD, possibly due to occasional overexpression of myeloperoxidase and reactive oxygen species, thereby worsening tissue injury and advancing atherosclerosis [26]. The primary pathogenesis of CVD arises from thrombosis following the rupture of plaques. Previous research has found a significant presence of neutrophils in ruptured carotid artery plaques, indicating a correlation between the neutrophil count and susceptibility to plaque rupture [27].

### 4.2 HDL and atherosclerosis

In contrast to neutrophils, researchers have regarded HDL as a protective factor against atherosclerosis-related CVD [28]. Some studies even argue that HDL-C levels are not a specific causative factor for cardiovascular events but rather may serve as a risk marker [29]. The primary mechanisms for HDL against CVD involve its anti-inflammatory effects on the endothelium, anti-oxidative properties, reduction of intravascular lipid deposition through facilitated reverse cholesterol transport, and attenuation of atherosclerosis by inhibiting monocyte formation and subsequently suppressing the inflammatory response [30,31].

### 4.3 Lymphocytes and atherosclerosis

Lymphocytes contribute to a favorable outcome in CVD by regulating inflammatory responses and thus exerting a protective effect against atherosclerosis. This is primarily attributed to the inhibitory effect on atherosclerosis by regulatory T cells, a specific subtype of lymphocytes [32]. Earlier studies have also demonstrated that a lowered lymphocyte count can be used as a surrogate indicator for physiological stress, stress-related CVD, and systemic hemodynamic instability induced by myocardial ischemia mediated by the release of cortisol [33]. Elevated cortisol levels lead to a decrease in lymphocyte production [34].

### 4.4 NHR & NLR and CVD

Multiple potential mechanisms can explain the connection between high NHR and NLR and the increased risk of CVD. NHR, involving inflammation and dyslipidemia, is commonly employed in clinical settings for CVD risk assessment. It acts as a predictive tool for determining the risk of coronary heart disease and assessing the severity of coronary stenosis or its prognosis [8]. NHR has also been utilized as a risk factor for the severity of ischemic stroke [35]. NLR is also related to the risk of CVD, including CAD and stroke. A meta-analysis including 38 studies (*n* = 76,002) showed that high NLR was significantly associated with the risk of CVD with pooled ORs of 3.86 (95 % CI: 1.73, 8.64). [5] Although the combination of NHR and NLR showed a stronger association with ischemic stroke [35], the joint effect of NHR and NLR on CVD did not demonstrate a stronger association than NHR alone in our study.

### 4.5 NHR & NLR and all-cause mortality

Compared to the impact of NLR on mortality, there is limited evidence suggesting a beneficial effect of NHR on all-cause mortality. A 7-year cohort study indicated that elevated NHR was considered an independent predictor of all-cause mortality in the general population. In this study, univariate Cox regression analysis demonstrated that the highest NHR quartile was associated with an increased risk of all-cause mortality (HR = 1.48, 95 % CI: 1.20–1.83) compared to the lowest one [12]. However, in our study, high NHR was not significantly associated with the risk of all-cause mortality after adjusting for relevant confounders. Elevated HDL levels are associated with an increased risk of mortality, with HDL levels demonstrating a J-shaped dose-response relationship with all-cause mortality [36]. Another cohort study demonstrated that higher NLR levels were associated with a high risk of all-cause mortality in the US general population, results consistent with our study [37]. This association is primarily attributed to the correlation of NLR with various adverse outcomes, including malignancy, CVD, and chronic inflammatory diseases [[38], [39], [40]].

There were several strengths in our study. Firstly, we conducted direct comparisons of the relationship between NHR and NLR and the risk of CVD and all-cause mortality in Taiwan, within a community-based prospective study. We identified and established appropriate cutoff values specific to the Taiwanese population and also adjusted for confounding factors using statistical analysis, thereby enhancing the robustness of the results. However, there were also some limitations in our study. First, our study, constrained to a single community without a large sample size and strong population representation, may lead to minor selection bias. Additionally, relying on observational data prevents the inference of causal relationships. Second, this study excluded patients with pre-existing CVD, thus limiting the investigation into the risk associated with secondary prevention. Thirdly, the association between dynamic changes in NHR and NLR ratios and the prognosis of CVD and all-cause mortality remains unclear, as only one blood sample was collected at the beginning of the study without subsequent serial measurements.

## 5 Conclusion

This prospective cohort study demonstrates the potential application of NLR and NHR as cost-effective and readily available markers for predicting the risk of CVD and mortality. High NHR was associated with the risk of CVD, while high NLR was related to the risk of mortality in the community-based population. However, the joint effect of NHR and NLR did not provide additional benefits in terms of relevant outcomes in the general population.

## Data availability

The datasets generated or analyzed during the current study are available from the corresponding author on reasonable request.

## CRediT authorship contribution statement

**Shih-Ming Chuang:** Writing – review & editing, Writing – original draft, Methodology, Conceptualization. **Sung-Chen Liu:** Supervision, Methodology. **Ming-Nan Chien:** Supervision, Methodology. **Chun-Chuan Lee:** Supervision, Methodology. **Yuan-Teh Lee:** Methodology, Conceptualization. **Kuo-Liong Chien:** Writing – review & editing, Supervision, Methodology, Conceptualization.

## Declaration of competing interest

The authors declare that they have no known competing financial interests or personal relationships that could have appeared to influence the work reported in this paper.
